# Smart and Sustainable: A Global Review of Smart Textiles, IoT Integration, and Human-Centric Design

**DOI:** 10.3390/s25237267

**Published:** 2025-11-28

**Authors:** Aftab Ahmed, Ehtisham ul Hasan, Seif-El-Islam Hasseni

**Affiliations:** Faculty of Engineering, Free University of Bozen-Bolzano, 39100 Bozen-Bolzano, Italy; aftab.ahmed@student.unibz.it (A.A.); seifelislam.hasseni@unibz.it (S.-E.-I.H.)

**Keywords:** smart textiles, IoT, human-centric design, wearable sensors, sustainability, e-textiles, biodegradable materials

## Abstract

Smart textiles are emerging as transformative modern textiles in which sensing, actuation, and communication are directly embedded into textiles, extending their role far beyond passive wearables. This review presents a comprehensive analysis of the convergence between smart textiles, the Internet of Things (IoT), and human-centric design, with sustainability as a guiding principle. We examine recent advances in conductive fibers, textile-based sensors, and communication protocols, while emphasizing user comfort, unobtrusiveness, and ecological responsibility. Key breakthroughs, such as silk fibroin ionic touch screens (SFITS), illustrate the potential of biodegradable and high-performance interfaces that reduce electronic waste and enable seamless human–computer interaction. The paper highlights cross-sector applications ranging from healthcare and sports to defense, fashion, and robotics, where IoT-enabled textiles deliver real-time monitoring, predictive analytics, and adaptive feedback. The review also focuses on sustainability challenges, including energy-intensive manufacturing and e-waste generation, and reviews ongoing strategies such as biodegradable polymers, modular architectures, and design-for-disassembly approaches. Furthermore, to identify future research priorities in AI-integrated “textile brains,” self-healing materials, bio-integrated systems, and standardized safety and ethical frameworks are also visited. Taken together, this review emphasizes the pivotal role of smart textiles as a cornerstone of next-generation wearable technology, with the potential to enhance human well-being while advancing global sustainability goals.

## 1. Introduction

### 1.1. Historical Evolution of Smart Textiles

Smart textiles, often referred to as electronic textiles (e-textiles) or intelligent fabrics, represent a new generation of textiles that extend the role of fabrics beyond aesthetics or protection into dynamic, interactive systems. Unlike conventional textiles, they actively sense, process, and sometimes actuate responses to environmental or physiological changes. Their architecture typically integrates conductive fibers, textile-based sensors, actuators, localized power sources, and embedded data processing and communication units, often interconnected through Internet of Things (IoT) platforms [1,2,3].

This inherently multidisciplinary field combines advances in materials science, electronics, computer science, and human-centered design, reflecting the convergence of form, function, and usability. In addition to comfort and aesthetics, smart textiles emphasize wearability, unobtrusiveness, and robustness in real-world use. Challenges remain, such as signal degradation under stretching and laundering, integration of stable power supplies, and long-term mechanical resilience, but continuous innovation is addressing these issues [4,5]. Increasingly, sustainability has also emerged as a critical dimension, as embedded electronics raise concerns regarding recyclability and e-waste. Recent developments, such as Silk Fibroin Ionic Touch Screens (SFITS), exemplify efforts to pair high-performance sensing with biodegradable substrates, pointing toward a future of environmentally responsible smart textiles [2,6].

The evolution of smart textiles began with efforts to embed electronic devices in clothing. At first, this process relied on rigid components sewn into fabric, which frequently resulted in garments that were uncomfortable for daily use [1]. The miniaturization of electronics, alongside breakthroughs in conductive fibers, polymers, and nanostructured coatings, transformed these concepts into fabrics that could embed sensing and actuation directly into their structure [7].

The advent of ubiquitous computing and IoT marked a second phase, where smart textiles evolved from isolated prototypes into distributed sensing platforms capable of real-time connectivity with edge or cloud systems [3,8]. With this shift, priorities moved from demonstrating feasibility to emphasizing durability, washability, and human-centric design. Increasingly, research has aimed at ensuring unobtrusiveness and user comfort while expanding functionality. More recently, the integration of Artificial Intelligence (AI) into textile-based systems has opened new directions, allowing not only continuous monitoring but also adaptive and predictive feedback for healthcare, sports, and other applications [2,6].

The remainder of this review is structured as follows: Section 2 provides a detailed analysis of the core technologies, focusing on conductive fibers, textile-based sensor types, and communication protocols essential for IoT integration. Section 4 highlights the cross-sector applications of smart textiles in fields such as healthcare, sports, defense, fashion, and robotics. Section 4 addresses the critical intersection of human-centric design and sustainability considerations, including challenges related to durability, washability, e-waste, and emerging design-for-disassembly strategies. Finally, Section 5 concludes the review by summarizing key findings and outlining future research priorities.

### 1.2. Pervasive Impact: Applications of Smart Textiles Across Industries

Smart textiles are impacting diverse industries, where their embedded sensing and IoT-enabled connectivity enable new forms of monitoring, interactivity, and feedback:

- Healthcare: Textile-based ECG electrodes, respiration monitors, and thermistors support continuous monitoring of vital signs, remote patient care, and rehabilitation. Smart bandages equipped with sensors can track wound healing and deliver controlled therapy, advancing personalized and preventive medicine [2,3].
- Sports & Fitness: Garments integrating sensors capture muscle activity, gait patterns, and joint load, providing real-time performance feedback and personalized training while reducing the risk of injuries [8].
- Defense & Security: Military uniforms with integrated physiological sensors, situational awareness systems, and adaptive camouflage technologies support soldier safety and operational efficiency. These systems reduce the burden of carrying separate devices by embedding functionality directly into fabrics [1,4,5].
- Fashion & Wearables: Interactive garments combine functionality with aesthetics. Examples include fabrics that change color or emit light in response to stimuli, as well as clothing that integrates wellness tracking without compromising comfort or style [7,9,10].
- Other Emerging Sectors: Applications extend to automotive interiors (smart seat fabrics for comfort and safety monitoring), smart homes (responsive furnishings), and robotics (soft fabric-based tactile skins enabling human–robot interaction) [3,6].

Across all these domains, embedded sensors and connectivity are essential, enabling not just passive data collection but predictive analytics and adaptive feedback. Taken together, these advances position smart textiles as a cornerstone of next-generation human–technology interaction.

However, the widespread adoption of smart textiles is contingent upon addressing significant barriers. These include technical and logistical challenges such as durability and washability, alongside critical considerations for sustainable design, energy consumption, and end-of-life management, which vary across different industry sectors. The following Table 1 summarizes the key applications, benefits, challenges, and sustainability considerations across these domains.

**Figure 1:**
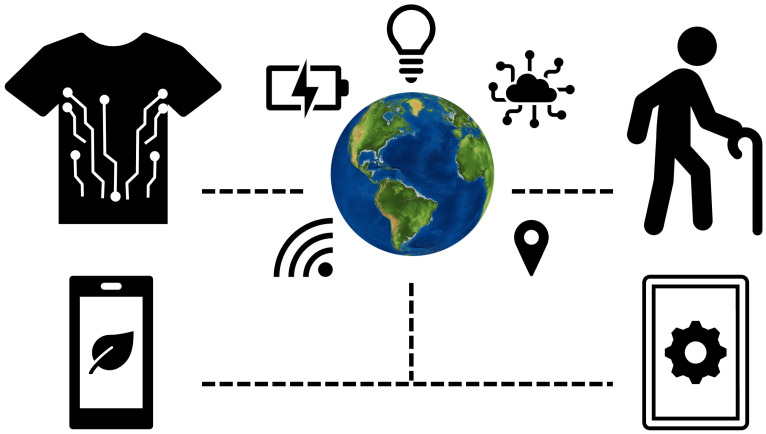
Graphical abstract summarizing the comprehensive scope of this review, highlighting the convergence of sensing technologies, IoT integration, human-centric design, and sustainability considerations.

Overall, this review brings together recent developments in smart textiles with a particular focus on sensing technologies, IoT integration, human-centric design, and sustainability considerations, highlighting their crucial role in advancing technology and improving human experiences. It covers material innovations such as conductive fibers and biodegradable substrates, system-level approaches including data processing and connectivity, as well as cross-sector applications in healthcare, sports, defense, fashion, and robotics. In addition, this review critically discusses challenges related to durability, washability, energy efficiency, and e-waste, and explores emerging strategies for sustainable design. An overview of the scope and structure of this review is presented in the graphical abstract (Figure 1), which highlights the convergence of sensing technologies, IoT integration, human-centric design, and sustainability considerations.

### 1.3. Literature Search and Selection Methodology

To ensure a comprehensive and up-to-date analysis, the literature search focused primarily on publications from 2018 to 2025, capturing the most recent advancements in the field. The selection process followed a systematic approach across three major scientific databases: Web of Science, Scopus, and Google Scholar.

The core search strategy utilized a combination of keywords related to the review’s main thematic areas, including:

- (“Smart Textiles” OR “E-textiles” OR “Wearable Electronics”)
- AND (“Sensing Technologies” OR “Pressure Sensor” OR “Strain Sensor”)
- AND (“IoT Integration” OR “Communication Protocols” OR “Wireless”)
- AND (“Sustainability” OR “Green Manufacturing” OR “E-waste” OR “Recyclability”)

Inclusion and Exclusion Criteria:

The initial search results were refined based on the following criteria:

- Inclusion: Priority was given to peer-reviewed journal articles and high-impact review papers that explicitly covered the convergence of smart textile materials/sensing and IoT integration, alongside detailed discussions of human-centric design or sustainability challenges.
- Exclusion: Papers that were outside the defined timeframe, restricted to applications irrelevant to textiles (e.g., rigid electronic devices only), or conference abstracts without full paper publication were generally excluded.

This systematic process ensured that the review is grounded in the most current, relevant, and authoritative literature supporting the core themes of sensing, IoT, and sustainability.

## 2. IoT Integration in Smart Textiles: Communication and Data Flow

### 2.1. Overview: Architecting Connectivity in Wearable Systems

The integration of smart textiles into the Internet of Things (IoT) ecosystem requires a seamless architecture that connects sensing, processing, and communication. At the foundation are textile-embedded sensors that capture physiological and environmental data such as temperature, strain, or bioelectrical signals. These signals are relayed to lightweight microcontrollers or edge devices that perform initial data processing and filtering before transmission. Connectivity modules embedded within the fabric link the system to external platforms, either locally (e.g., smartphones, gateways) or globally through cloud-based services [11].

To reduce delay and power consumption, edge computing is increasingly used within textile-integrated systems, enabling real-time analysis without continuous cloud dependency. This approach not only conserves bandwidth but also strengthens privacy by limiting the transfer of raw personal data. Energy efficiency remains a central concern; low-power communication and optimized processing architectures are essential to enable prolonged operation without frequent recharging. In parallel, the development of human-centric wireless sensor networks ensures that such systems remain unobtrusive, comfortable, and socially acceptable in daily use [12].

### 2.2. Common Communication Protocols for Smart Textile IoT

Selecting the appropriate communication protocol is critical, as it determines the reliability, efficiency, and scalability of textile-based IoT systems. Four protocols dominate current applications, each suited to distinct use cases:

Bluetooth Low Energy (BLE) offers short-range, low-power communication, making it ideal for personal area networks where data is transmitted to a nearby smartphone or wearable hub. BLE is particularly effective for fitness monitoring and healthcare applications requiring real-time feedback but limited range.

Zigbee supports mesh networking, allowing multiple textile sensors to connect in scalable networks. This makes it suitable for scenarios where robustness and fault tolerance are needed, such as team-based sports monitoring or collective worker health systems in industrial environments.

LoRaWAN provides low-power, long-range communication over several kilometers, making it well suited for remote health monitoring, agricultural applications, or scenarios where continuous smartphone connectivity is not feasible. Its lower data rates limit bandwidth-intensive applications but enable ultra-low power operation.

Wi-Fi and NFC are used in specific contexts: Wi-Fi enables high data rate transmission and direct internet connectivity, but at the expense of higher energy demands, limiting its textile integration. NFC, on the other hand, supports short-range, low-energy interactions, useful for authentication or data transfer in secure textile applications.

The performance trade-offs inherent in these standards highlight the necessity of application-specific protocol selection. Table 2 provides a comparative summary of the key technical characteristics, illustrating how factors like range, data rate, power consumption, and security govern suitability for specific smart textile applications.

**Figure 2:**
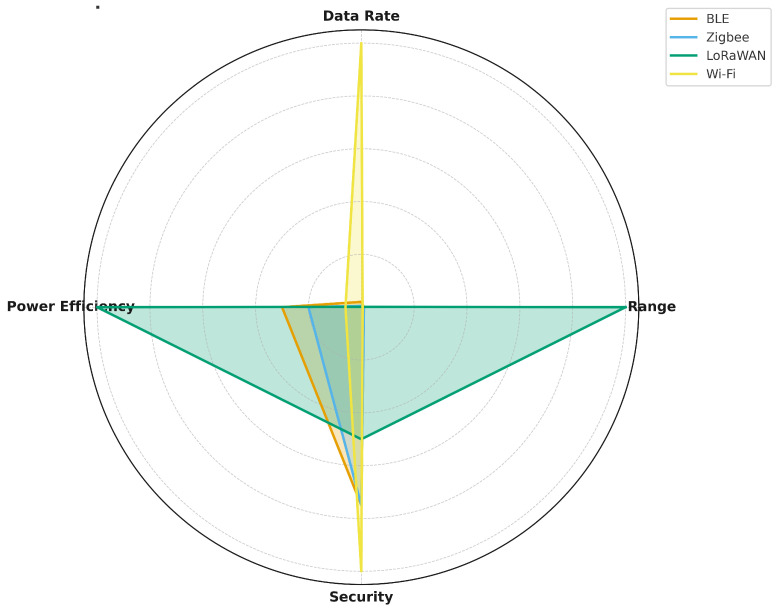
Trade-offs in key characteristics for IoT communication protocols in smart textiles. The visualization underscores the critical need for application-specific protocol selection based on balancing requirements like data throughput, power efficiency, and range.

A comparative evaluation of these protocols in terms of range, data rate, power efficiency, and security is shown in Figure 2. The radar chart highlights the trade-offs that must be considered: while Wi-Fi excels in data throughput and security, BLE and LoRaWAN are superior for energy efficiency, and Zigbee provides the advantage of scalable mesh networking. Thus, protocol selection should be guided by the intended application rather than a one-size-fits-all approach. As highlighted in Table 2, a critical trade-off analysis must be performed: protocols like Wi-Fi excel in data throughput and security for high-bandwidth needs, while others like BLE and LoRaWAN are superior for applications requiring low energy consumption and long-term autonomy. The selection must therefore be guided by the intended application requirements rather than a one-size-fits-all approach.

### 2.3. Challenges in Textile-Based Communication Networks

Although IoT integration unlocks unprecedented possibilities for smart textiles, it also introduces significant challenges that must be addressed to ensure reliability and user acceptance.

Power Consumption and Management: Textile-based devices rely on miniature batteries or energy harvesting systems, but frequent recharging can reduce usability. Advances in flexible photovoltaics, thermoelectric harvesters, and energy-aware protocols are being investigated to mitigate this limitation [13].

Data Security and Privacy: Smart textiles collect highly sensitive biometric and physiological data, making privacy a critical commercialization bottleneck. Beyond implementing standard robust encryption and secure key exchange protocols, protecting this information requires adopting fine-grained access control models and ensuring compliance with stringent regulatory frameworks (e.g., HIPAA, GDPR). Furthermore, to enable private data analysis, emerging cryptographic techniques are necessary, such as homomorphic encryption (allowing computation on encrypted data) and utilizing decentralized architectures like federated learning and blockchain-based verification to maintain data integrity and user control [14,15]. Without such comprehensive and advanced safeguards, the adoption of textile IoT systems could face significant societal resistance.

Signal Interference and Attenuation: The textile environment and constant body movements pose unique challenges for wireless communication. Antennas embedded in fabrics are prone to bending, stretching, and detuning, which can degrade signal quality. Adaptive modulation and error correction, as well as textile-optimized antenna design, are emerging as solutions.

Durability and Flexibility of Modules:Embedding rigid electronic modules into flexible fabrics often compromises comfort and longevity. Research into stretchable antennas, conductive yarns, and encapsulation technologies seeks to enhance durability without sacrificing textile properties. Standardized testing for washability and mechanical robustness will be critical to validate these innovations.

Together, these challenges underscore that the success of IoT-enabled smart textiles depends not only on sensor and communication performance but also on interdisciplinary advances in energy management, security, materials science, and human-centered design.

Looking ahead, IoT integration in smart textiles is expected to evolve beyond conventional communication toward more intelligent, decentralized systems. Future fabrics will not only transmit data but also embed edge intelligence, enabling localized analysis through lightweight AI algorithms and distributed “textile brains”. Such architectures reduce dependence on centralized cloud infrastructures, minimize latency, and enhance privacy while supporting adaptive, user-centered applications. As communication protocols mature and converge with advances in materials and embedded intelligence, IoT-enabled smart textiles will form the backbone of next-generation wearable ecosystems, seamlessly linking the present state of connectivity with the broader research directions outlined in Section 6.

## 3. Sensor Integration into Textiles: Technologies and Considerations

Recent advancements have sparked considerable interest in textile-based sensors for their versatile applications. These sensors are particularly well-suited for wearable electronics due to the inherent properties of textiles, which are flexible, lightweight, and conformable to the human body. This makes it possible to seamlessly integrate sensors into clothing for continuous and unobtrusive monitoring of physiological data like heart rate, respiration, and movement. Unlike rigid sensors, textile-based alternatives provide enhanced comfort and wearability, which can improve user compliance and enable extended use [16,17]. Beyond physiological monitoring, these sensors are valuable for applications such as pressure mapping in smart textiles and posture tracking for healthcare and sports analysis. A key benefit is their ability to be incorporated at various stages of textile production, including during the manufacturing of fabric, fibers, yarn, or in the knitting process. This seamless integration offers a cost-effective and scalable manufacturing solution [18].

Nevertheless, textile-based sensors possess specific limitations worthy of consideration. Challenges such as mechanical stress from bending or stretching, repetitive laundering, and exposure to environmental factors can lead to the loss of sensor function over time [19]. Another significant barrier is the intricate process of signal filtering, noise reduction, and data analysis required for accurate interpretation [20]. The large datasets generated by textile-based sensors require sophisticated algorithms for signal processing and pattern recognition to extract useful information [21]. Despite their potential, these sensors often struggle to match the sensitivity and specificity of traditional rigid sensors, which can compromise their overall performance [22,23].

### 3.1. Types of Textile-Based Sensors

The performance and integration of smart textiles are fundamentally determined by the type of sensing element used and its structural relationship to the fabric. Table 3 summarizes the key characteristics, methods, advantages, and limitations of the major textile-based sensor categories, highlighting the trade-offs inherent in their design and application.

**Figure 3:**
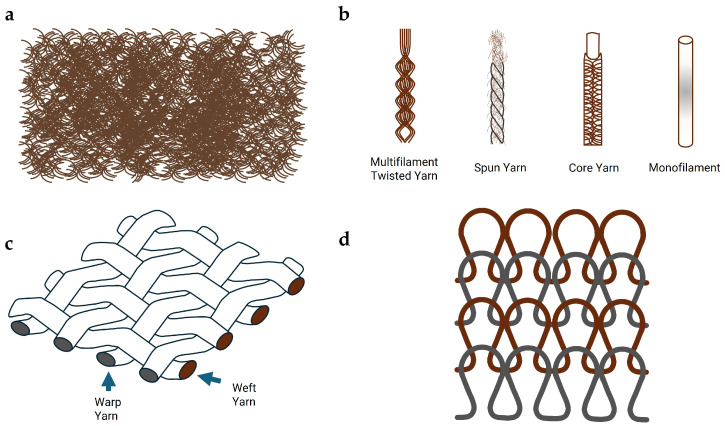
Illustrative comparison of textile sensor integration methods: (**a**) Fiber-based sensors, where the entire fiber is the sensing element; (**b**) Yarn-based sensors, where functional yarn based on multifilament, spun, core yarn or monofilament; (**c**,**d**) Fabric-based sensors, demonstrating how yarns are assembled (woven or knitted) to create a large-area sensing matrix.

The structural hierarchy of smart textile components, from the fundamental fiber level up to the final woven or knitted fabric, dictates their final application and performance. Figure 3 provides a visual representation of these different integration approaches.

#### 3.1.1. Fiber-Based Sensors

**Figure 4:**
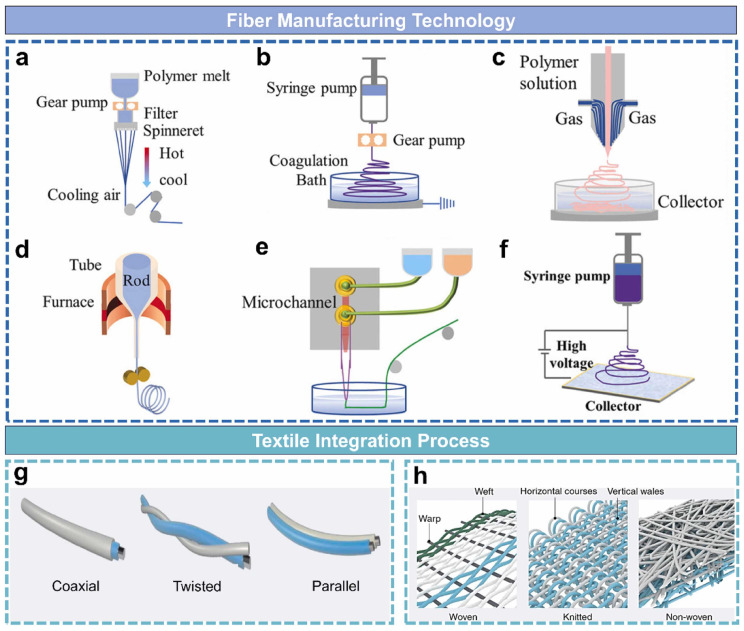
Schematic diagram of the manufacturing process of smart fibers and integration in textiles. (**a**) Melt spinning. (**b**) Wet spinning. (**c**) Blow spinning. (**d**) Thermal drawing. (**e**) Microfluidic spinning. (**f**) Electrospinning. (**g**) Various fibers in different arrangements form yarns. (**h**) Yarns are woven, knitted, or nonwoven ways to integrate textiles. Reproduced with permission from ref. [32], Copyright 2025, Elsevier.

In comparison to conventional silicon-based and thin-film sensors, fiber gives an excellent option for wearable sensors due to its superior flexibility, breathability, customizability, and adaptability to diverse anatomical regions [24,25]. Fiber-based sensors can be seamlessly incorporated into everyday apparel, including gloves, wristbands, elbow pads, knee pads, and shoe insoles [26,27,28]. Furthermore, fiber-based sensors can seamlessly integrate with a wide range of sensing fabrics, enhancing their versatility [29,30,31]. Products with fiber-based sensors allow for unrestricted movement while facilitating the accurate, real-time health monitoring resulting from human body deformation. These devices can monitor a wide range of personal digital health signals, such as biomechanical signals, biotemperature, biofluids, and breathing gas. Due to these exceptional advantages and broad applications, fiber-based sensors hold significant promise in developing compact, lightweight, cost-effective, and efficient personal digital health monitoring systems. A schematic overview of the manufacturing processes for smart fibers and their integration into textiles is shown in Figure 4 [32].

Conductive fibers are of materials with intrinsic electrical conductivity, including metals (e.g., pure silver wires, copper filaments, and stainless steel yarns) and conductive polymers such as polyaniline and PEDOT:PSS. These materials demonstrate conductivity due to their composition and do not require further plating or coating. Functionalized fibers are produced from non-conductive materials (e.g., nylon, polyester, cotton) that have been further coated, doped, or modified (e.g., chemical vapor deposition, ion implantation) to achieve electrical conductivity [33]. For instance, silver-plated nylon yarn achieves conductivity by the silver layer depositing onto nylon fibers. The silver plating provides conductivity to the yarn, despite the nylon core being non-conductive. A significant study [34] made a very extensible strain sensor using silver-plated nylon yarn. The sensor had exceptional sensitivity, with a gauge factor of 20,000, and was capable of detecting strains of up to 100%. In a similar study, [35], a flexible pressure sensor was fabricated using a silver-plated nylon yarn as the conductive electrode. The sensor exhibited an exceptional sensitivity of $2.32\times 10^{6}\;\;{\mathrm{kPa}}^{-1}$, highlighting its high responsiveness to pressure changes, and offered a wide pressure sensing range of around 40 kPa, allowing for versatile applications.

Conductive polymers, including polyaniline (PANI), polypyrrole (PPy), and PEDOT, have been thoroughly investigated for the development of conductive fibers and yarns. These polymers offer advantages such as flexibility, reduced weight, and simpler processing compared to metal fibers. Jia et al. [36] developed highly flexible electronic textiles coated with PEDOT via vapor phase polymerization for applications in wearable thermoelectric generators and strain sensors. The PEDOT coating consistently enveloped the curved surfaces of the textiles, demonstrating remarkable mechanical elasticity, electrical characteristics, and water resistance as a result of the strong in situ polymerization process. The PEDOT-coated textiles exhibited remarkably low sheet resistances, ranging from 24 to 155 Ω/sq, surpassing previously reported values. The strain sensors showcased an exceptional gauge factor of 54 at a strain of 1.5%, showing the highest value for textile-based strain sensors. Even when subjected to high strain levels of 10%, the strain sensors maintained a gauge factor of 2.

Functionalized fibers have also been developed by modifying traditional textile fibers like cotton, polyester, or nylon to enhance their electrical conductivity. Various methods, like coating, blending, or doping with conductive materials, can be used to enhance the electrical conductivity. Coating is a common method that stands out for enhancing the functionality of textile fibers. Methods like dip-coating, spray-coating, or chemical vapor deposition can be used to apply conductive materials, including metal nanoparticles, carbon nanomaterials, or conductive polymers, to the surface of the fibers. For example, Liu et al. [37] fabricated SWCNT-modified cotton threads through the dip-coating technique. Similarly, Reddy et al. [38] developed a flexible strain sensor by applying reduced graphene oxide to a polyester knitted elastic band.

#### 3.1.2. Yarns-Based Sensors

Melt spinning and solution spinning are the two main types of yarn manufacturing procedures used in industrial production. The process of melting a substance to a viscous fluid condition and then spinning, which is called melt spinning.

Integrating yarns with conductive elements offers a cost-effective alternative to expensive analytical instruments used in sports medicine and biomedical fields for physiological monitoring [2]. These conductive fibers can be spun into a continuous thread by creating an axial connection at the initial joint [39]. Composite yarns, which are formed by twisting together two or more individual yarns, have been used in various applications [40]. In a notable study, Lin et al. created composite yarns by combining Bamboo Charcoal (BC), a Phase Change Material (PCM), and Stainless Steel (SS). Their method, using a ring spinning frame, resulted in a core-shell structure. The core was made of BC/SS wrap yarns, while the shell was composed of BC roving and PCM [41]. A novel elastic warp-knitted fabric was developed by using the previously created complex yarns as weft yarns and combining them with elastic threads and polyester filaments as warp yarns. This fabric exhibits exceptional breathability and incorporates far-infrared and anion-releasing healthcare functionalities. The selection of this composite structure was crucial for maintaining the specific functions of the constituent yarns, particularly those related to photoelectric properties. This unique configuration gives the electrodes an appearance of independence while they remain functionally interconnected. Lotus et al. [42] successfully fabricated a composite yarn with a ZnO/NiO heterojunction structure. They accomplished this by modifying their electrospinning apparatus. The resulting yarn exhibited typical rectified current-voltage behavior and showed high sensitivity to ultraviolet (UV) light.

The advancement of electronic device reduction technology suggests that the integration of diverse electronic systems into yarns or textiles is going to become the prevailing trend in the future. The primary challenge lies in the fact that various electronic devices require distinct platforms with particular characteristics. To attain this objective, composite yarns will emerge as a suitable option.

#### 3.1.3. Woven-Based Sensors

The systematic yarn arrangement of woven structures enhances mechanical and performance stability, leading to their extensive use in the development of sensing materials. Diverse weaving patterns and adjustable sensor characteristics are enabled by the myriad combinations of interwoven units created by weft and warp yarns. Li et al. [43] investigated the impact of various parameters—including yarn type, weaving density, and patterns—on the sensing capabilities of a multilayer piezoresistive sensor. The sensor was constructed from woven functional fabric, interdigitated bottom electrodes, and PDMS encapsulation layers. The insulation provided by non-conductive polyester yarns to conductive silver-plated nylon yarns enhanced resistance variation within the woven network. This characteristic resulted in a wider sensitivity range, making it a better sensing layer option than a woven fabric made solely of conductive yarns due to its increased sensitivity. The study found that higher weaving density and longer float lengths improved the electrical contact performance when pressure was applied.

#### 3.1.4. Knitted-Based Sensors

Creating functional smart textiles often involves knitting, a technique valued for its ability to produce flexible, stretchable, and adaptable materials. Contemporary knitting methods allow for the seamless and customizable integration of materials on a large scale, enabling the creation of diverse 2D and 3D structures tailored to specific application requirements and operational modes. Several studies have explored the use of 2D knitting for smart textile applications. For instance, Chen et al. [44] developed a piezoresistive pressure sensor for pulse measurements using plain-knitted, double-plied conductive yarns. This design leveraged the highly elastic nature of the knitted structure to achieve a sensor with low hysteresis. Similarly, Lin and Seet [45] employed the intarsia knitting technique to embed a pair of textile electrodes made of conductive yarns, effectively sandwiching two piezoresistive films within the fabric. This sensor exhibited excellent linearity in its resistance change over a wide pressure range, up to 1 MPa. Despite the flexibility offered by 2D knitting, it can be restrictive in terms of complex designs and the integration of multiple sensors. In contrast, 3D knitting provides greater potential for creating hierarchical structures and integrating advanced functionalities. For example, Jiang et al. [46] used a 3D spacer-knitting technique with two distinct conductive yarns: one with lower resistance for surface electrodes and another with higher resistance for the central sensing layer. This approach facilitated multidirectional sensing (vertical pressure and lateral strain) of small mechanical loads (below ∼1 kPa/60%). However, a significant challenge with knitting for piezoresistive sensing layers is the inherently loose structure, which can cause friction between loops. This friction can negatively affect sensor performance by impacting signal quality and increasing hysteresis. This calls for meticulous optimization of engineering aspects related to material selection, yarn properties, and knitting parameters [44,47].

#### 3.1.5. Embroidery-Based Sensors

Embroidery, sewing, and stitching are among the most ancient additive techniques for decorating, joining, and layering fabrics. These methods involve the precise insertion of threads into a substrate, whether done manually or with a variety of specialized machines. They offer a great degree of design flexibility, making it easy to create functional applications by integrating aesthetic patterns onto textile substrates.

Parzer et al. [48] utilized a combination of sewing and embroidery to create interactive e-textiles with sensing capabilities. They incorporated piezoresistive yarns into items like a sofa and a pair of trousers to enable control of lights and smartphones. Both manual and machine-based methods were used to assemble orthogonally connected units and matrices. To enhance the force-sensing capabilities and aesthetic appeal, they manually embroidered yarn crossings over decorative, non-conductive embroidery patterns, effectively creating an interactive machine control panel. Since the foundational work of Post et al. [7], machine embroidery has been widely adopted for creating user interfaces and functional devices in e-textiles. A significant advantage of this approach is its high degree of automation, which allows for precise and reproducible pattern design and layer assembly [49,50].

Wang et al. built upon this technology by developing a toolkit for the rapid prototyping and computational design of functional textile interfaces. This system creates specialized sensing and feedback modules from commercially available yarns and fabrics, arranged in meticulously patterned and paired components. For example, machine embroidery has proven to be an effective method for anchoring or patterning cross-arranged yarn arrays, offering customized placement capabilities [51,52].

#### 3.1.6. Modification of Textile Substrates

Developing a sensor fiber network can be effectively achieved by directly modifying a textile substrate with conductive elements. Solution-based methods, such as coating (spray and dip coating) or in-situ deposition, are highly effective for this purpose. The performance and sensing reliability of these devices are significantly enhanced by ensuring strong coating adhesion to the substrate, which can be achieved through either chemical or physical bonding. The inherent physical characteristics of fibers and fabrics, specifically their curved and porous structure, present a significant challenge to achieving a uniform and stable coating deposition. This issue can be successfully addressed by selecting specific combinations of substrates and conductive nanomaterials that possess hydrophilic chemical properties. This approach facilitates a more even application of the conductive materials, leading to improved device performance. Various strategies have been explored to develop conductive textiles, often leveraging the intrinsic surface chemistry of certain materials. For example, the use of cellulosic textiles functionalized with MXene [53,54,55] or carbon nanotubes (CNTs) [56,57,58] is well-documented. These materials are favored for their excellent interfacial adhesion, a characteristic widely recognized by researchers. Developing these materials presents a particular challenge when working with hydrophobic textile substrates and solution-based fabrication methods. These techniques often necessitate the use of binding agents or surface modifications to achieve the desired conductivity, which can significantly increase the complexity of the fabrication process. An alternative approach, as demonstrated by Chen et al. [59], involved using an environmentally benign magnetic filtration cathodic vacuum arc deposition coupled with an ion beam technique. This method was successfully employed to create an exceptionally dense and uniform metal particle coating, improving the conductivity of a poly(aniline) (PANI)-modified cotton fabric. A common method for enhancing the adhesion of conductive coatings is to improve the substrate’s hydrophilicity and surface wettability. Plasma treatments, for instance, are frequently applied to yarns and fabrics to increase their hydroxyl groups, thereby promoting the anchoring of conductive layers [60,61]. This is a critical step in achieving durable and highly conductive textile materials. Several research teams have explored different methods for creating conductive textiles. Guo et al. [62] developed a straightforward and efficient technique that utilizes a physical self-assembly process. They employed bovine serum albumin, a natural adhesive, on a cloth substrate to improve the adhesion of GO/CNTs through hydrogen bonding and electrostatic interactions. Another approach, detailed by Clevenger et al. [63], involves an oxidative chemical vapor deposition (oCVD) method. This process polymerizes vapor-phase EDOT monomers, allowing for the direct deposition of uniform, conductive PEDOT coatings on various substrates in a single step, without the need for binders or surface pre-treatments. A mask was used to pattern these coatings, which demonstrated durability for up to 200 abrasion cycles. However, the washability of these devices needs improvement for practical wearable applications, and the manufacturing process may pose challenges for large-scale production due to the high-temperature chamber constraints.

Tian et al. [64] used a different strategy, incorporating hydrophilic polypropylene as a fiber core during the filament-forming process. This enhanced the wettability of a thermally bonded nonwoven fabric, facilitating the subsequent welding of carbon nanotube coatings. In another study, Doshi and Thostenson [65] utilized an acid-protonated polyelectrolyte, polyEthyleneImine (PEI), to create positively charged carbon nanotubes (CNTs). These CNTs were then attached to an aramid/polyester nonwoven fiber veil on a cathode using electrophoretic deposition under an electric field. The resulting piezoresistive sensor was found to be more stable and robust compared to carbon fiber and dip-coated samples. The environmental consequences of chemical byproducts from the solution-based creation of conductive substances are noteworthy [66]. Biomaterials like cellulose and silk are particularly popular for carbonization because they can be used to create highly conductive and responsive porous carbon materials. This method is both efficient and cost-effective, as it involves generating graphitic structures through pyrolysis.

Traditional thermal carbonization methods, however, require extremely high temperatures, often between 800 °C and 1000 °C or even higher. This demand for high heat results in poor energy efficiency due to significant energy consumption. In contrast, Laser-Induced Graphitization (LIG) has emerged as a prominent and more sustainable alternative. It’s a highly efficient method for directly creating conductive graphene patterns on organic substrates using laser pyrolysis, which is recognized as a more energy-conscious approach [67]. The LIG process yields porous microstructures that can be customized by manipulating machine settings like laser wavelength and scanning speed.

The ability to create in-situ laser-induced graphene without the need for binders is a major advantage for various flexible electronic applications, including but not limited to batteries, biomechanical sensors, and gas sensors [68]. This material has already proven effective in replacing traditional metal electrodes in textile-based piezoresistive pressure sensors and nanogenerators [69], showcasing its adaptability. It has also shown great potential for developing piezoresistive bending sensors using aramid yarns [70]. A key challenge, however, is the inherent mechanical instability and fragility of carbonized porous graphitic materials, which often require film encapsulation to make them suitable for use in wearable electronics.

### 3.2. Overview of Types of Sensors and Their Working Mechanism

There are two categories of sensors: active and passive. Active sensors can convert input energy into measurable output signals on their own, while passive sensors require an external power source for operation. Most textile-based sensors are passive, meaning they rely on external power for operation. Two types of textile-based wearable sensors being researched extensively are electromechanical sensors, which respond to mechanical forces, and electrochemical sensors, which detect chemical changes, with potential uses in real-time health and performance tracking. Electromechanical sensors generate an electric signal when mechanical force is applied, while electrochemical sensors detect chemical changes. Strain sensors and pressure sensors are used to measure the wearer’s respiration rate, pulse, muscle activity, and movements [71], which are influenced by physical force or body movement. On the other hand, a pH sensor can detect changes in sweat acidity [72], while a biomolecular sensor can recognize changes in glucose or lactate levels [73]. Researchers are creating smart clothing with built-in textile sensors to monitor body temperature, vital signs, and other biological changes in real-time, giving users ongoing health updates and personalized advice.

#### 3.2.1. Pressure Sensors

Fabric-based pressure sensors operate on three main principles: resistive, capacitive, and piezoelectric [74,75,76]. When subjected to a compressive mechanical force, these sensors produce a measurable electrical signal. They are often crafted by coating fabrics with conductive polymers [77] or by knitting them with conductive yarns [78]. The application of mechanical force causes a change in their resistance, capacitance, or results in the generation of an electric charge. The specific application of these sensors dictates how they are used to improve the functional and sensory characteristics of wearable technology. Over the last ten years, significant progress has been made in the creation of these sensors, leading to substantial improvements in their sensitivity, durability, and responsiveness. These sensors offer great flexibility, cost-effectiveness, and seamless integration into wearable devices, making them a versatile and indispensable technology in various fields.

Table 4 provides a quantitative comparison of the representative performance metrics, including sensitivity, working range, and response time, for the three main pressure sensing principles discussed in this section.

Piezoresistive sensors deform with compression, leading to variations in the contact area of the conducting material, which in turn causes changes in resistance. Piezoresistive-based pressure sensors are extensively researched compared to other wearable pressure sensors due to their straightforward structure, uncomplicated production procedure, low cost, and high durability. Furthermore, these sensors exhibit low power consumption and find extensive applications in the medical field [79] for monitoring vital signs and in sports [80] for performance tracking.

In their notable research, Zhang et al. [81] introduced a sophisticated textile-based piezoresistive sensor designed for the real-time detection of human motion. This innovative sensor was constructed using a multi-layered architecture, specifically by stacking several sheets of thiolated graphene@polyester (GSH@PET) textiles. Their findings demonstrated that these sensors are highly effective, boasting an impressive detection range spanning from 0 to 200 kPa, exceptional sensitivity, and a remarkably swift reaction time of 159 ms. In a separate investigation, a study [82] showcased a promising paper-based piezoresistive sensor that accurately monitors human physiological health, yielding encouraging results in the detection of vital signs. Furthermore, Tian et al. [83] contributed to the field by creating a pillow-shaped 3D hierarchical pressure-sensitive sensor. This device, engineered to track both breathing rate and prolonged human sleeping patterns, was fabricated using a combination of conductive silver-infused knitted fabric and a unique polypropylene fiber structure.

Piezoresistive sensors based on stitched technology have been fabricated utilizing stainless steel threads using a sewing machine [84]. When seamlessly integrated into clothing, sensors gain the ability to monitor human vital signs and muscle activity. This research explores an inventive approach to developing textile-based wearable sensors, leveraging the Force Sensing Resistor (FSR) principle. For example, one study by Tang et al. [85] detailed the fabrication of an elastic yarn made from carbon-based nanofibers, which was then knitted into a pressure sensor. Another notable development is a graphene-based resistive pressure sensor specifically engineered to precisely measure human pulse rates and elbow movements [86]. Similarly, Lou et al. [87] created a functional fabric by dip-coating polyester with Graphene Oxide (GO). This material exhibited a wide detection range and enhanced sensitivity, making it well-suited for precise pressure sensing in applications like plantar measurement and gait analysis. The simplicity of their design, along with their high responsivity and dependable repeatability, positions piezoresistive sensors as a strong candidate for future wearable sensing technologies, offering applications that range from tracking athletic performance to monitoring health metrics [88].

Capacitive sensors exhibit a variation in capacitance with the application of pressure. These sensors operate on the principle of a parallel-plate capacitor, wherein a dielectric substance is interposed between the plates. Applying an external force to a capacitor changes the distance between its plates, leading to a quantifiable shift in capacitance. Capacitive pressure sensors are particularly noteworthy for their high responsiveness, excellent sensitivity, and broad dynamic detection range. It is possible to boost the sensitivity of these sensors by adjusting the surface area, thickness, or material composition of the dielectric layer sandwiched between the conductive plates [89]. Over the past few years, there has been a significant body of research dedicated to creating textile-based capacitive pressure sensors specifically for use in wearable technologies. Lee et al. [90] exhibited sensors capable of being integrated into woven gloves and garments to facilitate wireless machine control and establish highly intuitive human-machine interfaces. A coating of polystyrene-block-butadiene styrene (SBS) polymer was applied to Kevlar fibers, which were then treated with silver nanoparticles (AgNPs). These treated fibers became highly conductive, demonstrating an impressive electrical resistance of 0.15 Ω^−1^ cm and robust bending stability. To create a capacitive sensor, a layer of poly(dimethylsiloxane) (PDMS) was used as a dielectric material, deposited on the surface of two of these conductive fibers that were oriented perpendicularly. The sensor exhibited high sensitivity and remarkable resistance to fatigue, withstanding more than 10,000 bending cycles. In a related study [91] conducted with a similar design, a sensor was integrated into a fabric to measure light finger pressure (less than 10 kPa) using a capacitance readout circuit. Separately, another approach involved making Twaron fibers conductive with the same SBS/AgNP combination. An extra layer of PDMS and glucose particles was then applied to form a microporous dielectric layer. This microporous structure enhanced the sensor’s deformation when pressure was applied, thereby improving the capacitive sensing performance.

Piezoelectric sensors have become increasingly prominent because of their utility, ease of electrical signal capture, straightforward design, and economical fabrication. These materials are capable of converting mechanical energy into electrical energy. When an external force deforms the material, its positive and negative charges separate, creating a potential difference that is proportional to the applied pressure. This unique property allows these materials to be used as both pressure sensors in wearable electronics and for energy harvesting.

Numerous research efforts have advanced the field of self-powered, multifunctional wearable sensors and electronic skins. In one such study, Yang et al. [92] created a piezoelectric pressure sensor for a shoe insole to monitor impact during walking, running, and jumping. The sensor, made from PolyDopamine (PDA)-modified barium titanate (BaTiO_3_) and PolyVinyliDene Fluoride (PVDF), demonstrated enhanced pressure sensitivity and a rapid response time of 61 milliseconds. This device proved effective in tracking various human movements, including those of the elbow and finger joints, when worn on the body. Similarly, Zhu et al. [93] developed a piezoelectric pressure sensor using electrospun PVDF nanofibers to accurately monitor biomechanical movements in the human finger, elbow, and foot. This fibrous sensor showed exceptional sensitivity to pressure changes, mechanical durability, and thermal stability. In another application, Wang et al. [94] presented a highly sensitive piezoelectric sensor for continuous blood pressure monitoring. By integrating the sensor into a wrist strap, they enabled direct skin contact for precise, real-time measurements. The device measured systolic blood pressure by analyzing pressure wave signals from the pulse, offering a continuous and highly accurate way to track blood pressure fluctuations. Furthermore, Kim et al. [95] explored the impact of Carbon NanoTubes (CNTs) on the dielectric and piezoelectric properties of a ceramic-epoxy nanocomposite. They found that adding multiwalled carbon nanotubes (MWCNTs) as conductive fillers significantly boosted the pressure sensitivity and output voltage of the piezoelectric sensors. A separate investigation highlighted that incorporating MWCNTs into PVDF also considerably improved the sensor’s conductivity and mechanical strength. This enhanced piezoelectric sensor was successfully applied to human skin to precisely monitor various motions, including tactile sensations, finger joint movements, and wrist flexion.

**Table 4:** Comparative Performance of Textile-Based Pressure Sensing Principles.

Sensing Principle	Representative Sensitivity	Typical Working Range	Response Time
**Piezoresistive**	High (e.g., up to $2.32\times 10^{3}\;{\mathrm{kPa}}^{-1}$)	Wide, from subtle to high pressure (0 to 200 kPa) [81]	Fast (e.g., 159 ms [81])
**Capacitive**	Excellent (adjustable by dielectric layer geometry)	Typically low-to-medium pressure (e.g., <10 kPa) [91]	Fast (e.g., tens of ms)
**Piezoelectric**	Good (Enhanced by CNTs/nanofillers [95])	Dynamic measurement of transient forces (e.g., impact monitoring)	Extremely fast (e.g., 61 ms [92])

#### 3.2.2. Strain Sensors

Strain sensors are capable of transforming physical deformations into electrical signals. The output signals may manifest as resistance, capacitance, or other forms, based on the specific type of sensor utilized. Strain sensors based on resistive and capacitive principles are frequently utilized in textile applications because of their straightforward fabrication and the convenience of embedding them [96,97]. These sensors are applicable in wearables for detecting various human movements and muscle activities [98], such as respiration [99], breathing, and pulse [100].

The resistance of a resistive strain sensor changes as a result of applied strain, which alters the length or area of the conductive textile electrode [101]. In contrast, a capacitive strain sensor sees its capacitance change when the area or distance between its two parallel textile electrodes, which are separated by a dielectric material, is altered by stretching [102]. Both types of sensors offer unique benefits. While resistive sensors are notable for their simple structure, capacitive sensors are often considered more sensitive, displaying quicker response times and less hysteresis. A significant challenge with capacitive strain sensors is that their parallel plate configuration also reacts to pressure, making it difficult to differentiate strain signals from pressure-induced ones. To address this, one might employ advanced signal processing algorithms to filter out pressure-related signals, thereby isolating those caused solely by strain. Textile-based strain sensors offer flexibility and comfort due to their integration with textiles, making them suitable for wearable applications [103]. Research indicates that applying a concentrated conductive polymer solution to textiles can render them rigid [104]. However, studies have shown that coated textiles can withstand specific elongation before experiencing a noticeable decline in mechanical properties. When cracks form in the conductive layer, the sensor’s accuracy and durability may be compromised. Maintaining conductivity during stretching ensures consistent and reliable sensing capabilities in textile-based strain sensors [105,106].

Wearable technology widely utilizes textile-based strain sensors for a variety of applications. For example, Li et al. designed a yarn-based strain sensor that can be seamlessly integrated into clothing to track various human movements. This particular sensor was constructed with a PolyUrethane (PU) core, a conductive multilayered sheath, and a thin outer layer of PDMS, making it both sensitive and flexible. This versatile sensor was then incorporated into medical textile bandages to demonstrate its ability to detect human pulse, finger flexion, and ambulation when worn on the wrist, finger, and leg, respectively. Additionally, the researchers integrated the sensor into gloves to successfully operate a robotic hand. A similar strain sensor, described by Luo et al., was designed to measure human joint movement, specifically at the elbow and knee. This sensor was produced using a screen-printing technique where silver nanowire ink was applied to a flexible textile. It demonstrated a wide operational strain range of 120%, high sensitivity, and significant durability, withstanding up to 2000 cycles. Such sensors hold great promise for use in athletic apparel for purposes such as rehabilitation, coaching, and injury prevention. In another study, a knitted strain sensor was created for a breathing belt using silver-plated nylon fibers. The mechanism behind this sensor involves the separation of conductive contact points in response to applied strain. Real-time testing on the human body to measure respiration rate under dynamic conditions showed encouraging results. All these research efforts highlight the immense potential of textile-based strain sensors in various fields [107,108,109].

#### 3.2.3. Temperature Sensors

Wearable sensors are gaining significant interest for their ability to provide an alternative approach to disease diagnosis and health monitoring through the collection of human physiological signals in a non/minimally invasive manner. Temperature is a crucial physiological marker in mammals, reflecting various physiological processes and overall health. Continuous and reliable temperature sensing is essential in health monitoring, playing a significant role in preventing, diagnosing, and tracking diseases like cardiovascular conditions, wound injuries, and other syndromes [110,111]. Wearable temperature sensors represent a significant advancement in temperature measurement, allowing precise detection in localized areas while adapting to dynamic changes in skin condition. In contrast to conventional rigid sensors, wearable alternatives offer key benefits like flexibility, biocompatibility, durability, and skin comfort, making them well-suited to meet the evolving demands of digital health monitoring [112].

Current practices in the development of textile-based temperature sensors primarily involve the integration of temperature-sensing fibers or yarns into fabrics through traditional methods such as stitching, knitting, or weaving. In recent years, a particular class of conductive polymers, Poly(3,4-ethylenedioxythiophene):Poly(styrenesulfonate)(PEDOT:PSS), has become a focal point of research interest. One notable approach involved creating a textile temperature sensor by dip-dyeing cotton thread in a semiconductive PEDOT:PSS solution [113]. This specific thread-type sensor was found to possess both high sensitivity and a broad operational temperature range, suggesting its suitability for integration into fabrics via sewing or weaving techniques. Another advancement in this field involved the creation of a PEDOT:PSS-based temperature sensor utilizing a Kapton (polyimide) film in conjunction with cotton textiles. This device was capable of detecting minute temperature changes, as low as 0.1 °C, and its flexible nature underscores its promise for a wide array of wearable applications [114].

Lugoda et al. [115] presented the development of temperature-sensing socks that were manufactured using specially designed knitting yarns. In their work, the authors emphasized that maintaining sufficient contact between the temperature sensor and the human skin is a critical factor to obtain accurate measurements. Building on this idea, a smart sock was later designed with the capability to provide continuous health-related data, such as monitoring foot temperature in patients [116]. Such data collection is particularly useful as it allows patients to make timely adjustments in their diet and medication routines, thereby improving health management outcomes. In parallel, researchers have been working to overcome challenges related to the relatively low mechanical strength of metallic fibers employed in temperature-monitoring systems. To address this issue, advancements in wrapping technology for metal yarns have been reported, showing significant improvements in both tensile strength and strain resistance, thus proving the reliability and efficiency of this method for wearable sensor applications [117]. In another notable contribution, Jung et al. [118] designed a textile-integrated temperature sensor for wearable systems by applying thermoelectric inks directly onto knitted fabrics. This approach resulted in a sensor that maintained excellent performance even when subjected to tensile stress, which is essential for ensuring long-term durability and consistent operation in wearable devices. Likewise, Li et al. [119] demonstrated a fabric-based temperature sensor by embedding metallic filaments into a woven fabric structure, offering a creative and effective strategy for integrating sensing functions directly within textiles. This woven sensor was incorporated into wearable platforms to showcase its ability to detect skin temperature during daily activities, highlighting its practical potential for real-world applications.

#### 3.2.4. Electrochemical Sensors

Wearable biosensors designed for clinical diagnostics and the monitoring of biological fluids such as sweat, blood, and saliva have significantly contributed to the emergence of the field commonly referred to as smart textiles. Within this context, flexible textile-based sensors function as analytical tools that are capable of identifying and measuring chemical signals generated by the human body, thereby supporting both disease detection and continuous health monitoring. The integration of chemical sensing elements directly into textile substrates not only provides real-time and valuable information about the wearer’s physiological state but also enhances the overall effectiveness of health-monitoring systems while maintaining the comfort and usability of garments.

Electrochemical wearable sensors have emerged as highly versatile analytical platforms for monitoring biomolecules in various biological fluids, as well as volatile substances generated by the human body. Among these, textile-integrated humidity sensors constitute a crucial subset within the field of electronic textiles, owing to their direct relevance in biomedical applications, most notably for wound management and therapeutic monitoring. The functional efficiency of such humidity sensors fundamentally depends on the use of fabrics that exhibit controlled electrical conductivity combined with pronounced responsiveness to water molecules. In a notable contribution, Zhou et al. [120] demonstrated the fabrication of a textile-based wearable humidity sensor by producing Single-Walled Carbon Nanotubes/Poly Vinyl Alcohol (SWCNT/PVA)) filaments through a wet-spinning process, which were subsequently integrated with cotton substrates. The resulting ultrastrong filaments enabled reliable monitoring of human perspiration, thereby confirming the sensor’s practical applicability. Similarly, Su et al. [121] reported the development of an impedance-type humidity sensor fabricated by spray-coating woven cotton fabric with an organic copolymer composed of methyl methacrylate and [3-(methacrylamino)propyl] trimethyl ammonium chloride. This design was strategically intended for tracking respiration dynamics before and after physical exertion, offering valuable insights into exercise-induced changes in breathing patterns. Furthermore, the physiological role of water molecules in modulating respiration has been well recognized, particularly through their effect on Relative Humidity (RH) near the oral and nasal cavities, which in turn influences the regulation and characterization of breathing processes [122].

Ma et al. [123] introduced a yarn-based humidity sensor that exhibits both high sensitivity and rapid responsiveness, making it particularly suitable for integration into wearable platforms aimed at monitoring human breath. Their approach involved wrapping a biaxial yarn twice around a copper wire to fabricate the sensing element, which was subsequently attached to a 3M face mask to enable real-time respiration tracking. To construct the functional yarn, copper wire was first wrapped with silk fibers, after which the composite yarn was coated with a polyimide layer and then sputter-coated with silver, thereby forming the final humidity-sensitive structure. Once incorporated into a smart face mask, the sensor successfully demonstrated the ability to capture and monitor the respiratory activity of the wearer. The unprecedented challenges of the COVID-19 pandemic have highlighted the urgent importance of Personal Protective Equipment (PPE), including masks, gloves, and gowns, with a sharp increase in their demand and relevance. In particular, the requirement for disposable barrier textiles, respirators, and protective masks has escalated due to intensified efforts toward infection prevention and control strategies during global health emergencies. Furthermore, the integration of electrochemical sensors into masks for real-time monitoring of critical health indicators such as breathing patterns and respiration rate has been shown to significantly improve health evaluations, support more accurate medical diagnoses, and aid in predicting recovery outcomes. This is especially valuable in cases where individuals are under self-isolation or quarantine, as emphasized in studies such as Morris et al. [124].

Textile-integrated electrochemical biosensors have emerged as essential tools for noninvasive health monitoring, primarily through the detection and analysis of biomarkers present in bodily fluids such as sweat. Since sweat provides a readily accessible medium that reflects the body’s physiological condition, its study has been particularly emphasized in relation to both athletic performance assessment and clinical applications for patients with specific health conditions. Within this field, pH sensing textiles have drawn considerable attention, as several investigations underline their effectiveness for sweat analysis and their potential incorporation into wearable platforms for continuous monitoring [125,126]. Beyond single-parameter detection, the advancement of multiplex systems has been demonstrated, such as the development of a wearable sweat-analyzing patch capable of real-time, simultaneous biomarker detection [127]. This patch, when positioned on the upper arm during physical activity, successfully identified a range of sweat constituents, including glucose, lactate, ascorbic acid, uric acid, Na^+^, and K^+^, thereby providing comprehensive physiological feedback. Similarly, Promphet et al. [72] presented a multifunctional electrochemical biosensor that could monitor both sweat pH and lactate concentration in tandem. To validate the sensor’s performance, extensive on-body experiments were carried out under dynamic conditions, reinforcing the sensor’s reliability and highlighting its suitability for wearable healthcare technologies. Bio-sensing textiles (BIOTEX), a European Union initiative, created a series of electrochemical sensors capable of measuring the pH and electrolyte concentration of sweat for prospective patient monitoring [128,129]. Integrating textile-based stretchable sensors into sportswear offers a valuable means to monitor athletes’ physiological conditions.

## 4. Sustainability, Environmental Impact and Technical Challenges of Smart-Textiles

### 4.1. Impact of Materials

The manufacturing of smart textiles poses significant sustainability challenges, particularly due to high resource consumption and various environmental impacts. Nonrenewable resources such as petroleum-derived polymers and rare-earth metals are frequently used as fundamental raw materials for smart textile production. Smart textiles often combine natural fibers, such as cotton, with synthetic fibers, including polyester. Cotton cultivation is associated with several environmental concerns, most notably water depletion and pesticide use. Although cotton is biodegradable and derived from natural sources, its production requires substantial amounts of water and agricultural chemicals.

The environmental burden associated with synthetic fiber manufacturing is also substantial, with polyester being one of the most significant contributors. Recent assessments from Textile Exchange indicate that global textile fiber production continues to grow, with fossil-based synthetics—particularly virgin polyester—representing the largest share and contributing considerably to greenhouse gas emissions during production [130,131]. These findings reinforce the concern that polyester production remains a major driver of the textile sector’s overall environmental footprint. In addition to the environmental pressures created by fiber production, the supply chain for rare-earth metals and related minerals presents another set of sustainability challenges. These materials are essential for the functionality of emerging smart textile technologies, especially for electronic components such as sensors and energy-storage devices. However, the extraction and processing of rare-earth elements are frequently linked to severe environmental degradation, including the release of toxic pollutants, the erosion of productive soils, and contamination of surface and groundwater resources [132]. Such consequences highlight the dual challenge of enabling technological advancement while ensuring responsible sourcing of critical materials.

The integration of electronic components into smart textiles further contributes to environmental pollution through chemical discharge, soil degradation associated with manufacturing operations, and water contamination resulting from improper waste disposal. Extraction and refinement processes also intensify global warming through emissions of greenhouse gases such as carbon dioxide, as well as air pollutants including sulfur dioxide and nitrogen oxides. Overall, these factors underscore the need for more sustainable material choices and improved production strategies within the smart textile industry.

### 4.2. Consumption of Energy

The development of smart textiles possessing conductive and energy-harvesting capabilities necessitates considerable energy investment and advanced production techniques. This energy-intensive production process poses a considerable barrier to sustainability. The production processes for advanced textiles and conductive materials, such as those incorporating carbon fiber, silver, and graphene, are characterized by high-temperature manufacturing stages and subsequent treatments that collectively impose substantial energy requirements. The root cause of this high energy demand often lies in the materials science processes, including: (1) High-Temperature Treatments, such as the carbonization and graphitization stages required to achieve high conductivity in carbon-based fibers; and (2) Vacuum or Specialized Atmosphere Environments, necessary for deposition techniques like Chemical Vapor Deposition (CVD) to integrate nanomaterials.

The integration of Phase Change Materials (PCMs) into textile structures, while improving thermal regulation and energy efficiency during the operational life of the product, simultaneously elevates the energy consumed during manufacturing; this increase is attributable to the necessity for incorporating specialized chemical additives and employing more complex fabrication techniques [133]. Similarly, the creation of smart conductive fabrics that utilize multi-walled carbon nanotubes (MWCNTs) alongside conductive polymers relies on energy-intensive procedures, with methods such as electrospinning and dip coating being particularly demanding [134]. Furthermore, the manufacture of yarn-based supercapacitors designed for integration into smart textiles involves considerable energy expenditure, especially during the critical phases of carbonization and other material processing steps, even though these yarns can achieve a notable power density of up to $27.5\;\;\mu W\;{\mathrm{cm}}^{-1}$ [135]. The production of carbon fibers specifically exemplifies this high energy demand, as it involves a series of thermally intensive steps including an initial stabilization stage conducted at temperatures ranging from 200 °C to 300 °C, which is then followed by a carbonization process that can approach temperatures of 1000 °C; the cumulative energy footprint for these processes is significant, with studies indicating a consumption range of approximately 295 to 740 kWh for every kilogram of carbon fiber produced [136].

### 4.3. E-Waste Generation

The primary e-waste challenge unique to smart textiles is their identity as highly complex composite materials, an inseparable blend of flexible textile polymers and rigid electronic components (conductive inks, microchips, sensors). The incorporation of smart devices into textile substrates frequently relies on robust connection techniques such as brazing, welding, soldering, or adhesive bonding, which inherently create highly stable, yet difficult-to-separate, composite joints. Substantial progress has been made to integrate components using established textile manufacturing techniques like weaving, sewing, stitching, and knitting [137]. However, these integration methods, whether chemical or mechanical, make the clean physical and chemical separation of the electronic fraction (e.g., metals, ceramics) from the textile fraction (e.g., polyester, cotton) practically impossible using conventional recycling infrastructure. A typical small electronic device can be composed of as many as forty distinct materials, and each of these materials often requires a highly specialized and unique recycling process due to the vast differences in their individual physical and chemical properties. Although a considerable number of these components hold intrinsic economic value, the majority also contain potentially hazardous constituents, which include various poisonous heavy metals and halogenated organic compounds [138]. The predominant recycling procedures currently in use commonly involve an initial shredding stage, a process which is often inadequate for achieving the complete and clean separation of these diverse material elements [139]. Furthermore, this inadequacy in the recycling infrastructure creates a significant risk that these dangerous compounds may be released into the surrounding environment. Compounding this issue, research has demonstrated that each individual washing cycle has the potential to result in the shedding and loss of approximately 1900 microfibers from a single garment [140], introducing an additional pathway for environmental contamination. Wäger et al. [141] indicated that radio-frequency identification (RFID) tags may interfere with traditional recycling methods for non-electronic goods. Government activities are implemented to encourage responsible recycling and disposal procedures among producers [142]. The efficacy of these solutions is constrained due to the disjointed nature of e-waste laws, resulting in uncertainty for both producers and consumers [143]. Consequently, researchers have suggested specific approaches to evaluate the End-Of-Life (EOL) of electronic equipment to improve recycling and reuse [144].

Factors influencing the e-waste recycling issue include increased electronic device production, hazardous materials in electronics, risks to human health and the environment, and the use of expensive materials. Waste-related difficulties can be significantly alleviated by the implementation of environmentally conscious design in manufacturing processes [145]. Implementing waste-preventive measures during the first development phases can be effective, particularly when e-textiles are not yet market-ready.

## 5. Approaches to Sustainability in Smart Textiles

The aim of complete sustainability in smart textile products presents a considerable challenge; however, the establishment of appropriate development guidelines for these materials can mitigate their overall environmental impact. This section discusses considerations that highlight simplicity, material efficiency, and environmental friendliness [146].

### 5.1. Eco-Friendly Raw Materials

Within the realm of smart textiles, traditional functional materials—encompassing conductive polymers, carbon-based nanomaterials, and various metal-based compounds—are extensively utilized to achieve specific and desired performance characteristics; however, the significant environmental ramifications associated with the production of these advanced textiles are primarily rooted in the heavy reliance on nonrenewable resources, the implementation of energy-intensive manufacturing methodologies, and the incorporation of materials that are not readily recyclable, all of which are frequently employed throughout the fabrication process. As substantiated by several research investigations, a strategic transition towards the adoption of biodegradable and recyclable material alternatives presents a viable pathway to substantially mitigate these adverse environmental impacts [147].

### 5.2. Renewable and Biodegradable Fibers

Non-biodegradable synthetic fibers, such as polyester and nylon, pose a significant environmental threat. Their production process generates significant carbon emissions due to the reliance on fossil fuels. The energy-intensive manufacturing process of one kilogram of polyester, requiring 125 MJ, leads to the emission of about 14.2 kg of CO_2_ per kilogram, directly contributing to greenhouse gas emissions [138]. Within the sphere of sustainable materials, cellulose-based fibers—encompassing natural varieties such as cotton and linen alongside regenerated forms—present themselves as renewable and biodegradable options, while also exhibiting a significant compatibility with various conductive substances, thereby facilitating their application in a diverse range of functionalized textile treatments and coatings. Particularly, Plant-derived Cellulose NanoCrystals (CNCs) are considered exceptionally well-suited for integration into flexible electronic textiles, not only because of this inherent compatibility but also due to their superior mechanical characteristics, which are critical for enabling enhanced device performance. Demonstrating a remarkable Young’s modulus in the range of 110 to 220 GPa and a tensile strength varying from 1 to 10 GPa, these plant-derived nanocrystals possess mechanical properties that are often likened to those of high-strength materials like Kevlar fiber [148]. Furthermore, when CNCs are combined with silver nanowires to form composite structures, the resulting material achieves an outstanding electrical conductivity of 104 S/m and exhibits exceptional thermal stability, maintaining its integrity at temperatures approaching 250 °C, which collectively render these composites highly appropriate for demanding applications within the field of wearable electronics [149]. The fungus-derived substance ’mycelium’ is gaining recognition for its substantial environmental benefits, attributed to its minimal water and energy requirements during production. Mycelium breaks down in soil within four to six weeks, making it a swift and sustainable raw material specifically suited for eco-friendly textile processes [150]. Graphene-collagen composites offer a promising alternative due to their exceptional flexibility, conductivity, and biodegradability, degrading in under 60 h [151].

### 5.3. Biodegradable Plant-Based Polymers for Coatings

Within the sphere of sustainable materials, renewable polymers such as PolyLactic Acid (PLA) and PolyHydroxyAlkanoates (PHA) serve as environmentally benign and biodegradable substitutes for conventional plastics like polyethylene and polypropylene; for instance, conductive fabrics that have been coated with PLA demonstrate not only enhanced mechanical flexibility but also a considerable tensile strength that typically falls within the range of 50 to 70 MPa, coupled with an elongation at break between 6 and 10%. A further significant advantage of PLA is its inherent biodegradability, particularly within industrial composting environments, which actively contributes to waste reduction strategies as the material undergoes decomposition over a period of approximately 6 to 12 months following its disposal. The notable tensile strength and flexibility characteristics inherent to PLA increase its suitability for various applications that demand long-term durability and structural integrity, while conversely, the comparatively faster biodegradation rate exhibited by PHA presents distinct benefits for the creation of products that are designed for single-use or temporary applications [152].

### 5.4. Green Manufacturing

Sustainable production embodies the principles of minimizing water and energy consumption, maintaining the material’s essential properties across its entire lifespan, and ensuring its capacity for recycling once its primary service life has concluded.

A crucial comparison exists between fiber formation techniques: the melt spinning technique involves the extrusion of molten, fiber-forming substances through a specialized spinneret, followed by a solidification stage that typically employs cold-air quench ducts. A significant advantage of this approach is its solvent-free nature, which precludes the generation of associated volatile organic compounds (VOCs) and circumvents complex solvent recovery or wastewater treatment challenges. This establishes melt spinning as a more favorable option compared to alternative methods like wet-spinning and dry-spinning, which necessitate the use of solvents and require dedicated, energy-intensive recovery systems to mitigate environmental discharge [138]. Quantitatively, wet spinning processes typically have a larger environmental impact due to the energy and infrastructure required for solvent recovery, despite having a lower energy demand for the initial heating phase compared to the high temperatures needed for polymer melting in melt spinning.

The introduction of conductive nanomaterials, including graphene and carbon nanotubes (CNTs), into textiles can be accomplished at the fiber-spinning stage by dispersing them directly within the polymer melt prior to extrusion; this process yields conductive fibers or filaments upon the extrusion and solidification of the composite material. This particular spinning methodology is notably applicable and effective for the production of common fibers such as those based on polyethylene terephthalate (PET) and polyamide (nylon). Moreover, in the specific context of developing e-textiles via melt spinning, the polymer melts of polypropylene (PP) and polyethylene (PE) can also act as foundational matrices, enabling the successful fabrication of ultrafine fibers [153].

Inkjet printing has emerged as a predominant and highly versatile methodology within the field of flexible printed electronics, providing a notably cost-efficient and high-resolution pathway for the fabrication of complex multi-layer devices. This technique facilitates the deposition of a wide array of functional materials, including insulating, semiconductive, and conductive substances, which can be strategically combined in various sequences to produce sophisticated heterostructures; these structures possess meticulously tailored properties that are engineered to enhance overall performance and to introduce a diverse range of functionalities. Consequently, inkjet printing stands as both a sustainable alternative and a complementary technique to the established process of multicomponent melt-spinning used for the production of multimaterial fibers. Presently, wearable devices encounter challenges related to reliability and manufacturing that can be effectively addressed through the implementation of printing technology in their production. The method provides a non-impact and maskless approach for the deposition of controlled quantities of materials with precision, speed, and reliability [138].

### 5.5. Electronic Waste Recovery and Treatment

E-waste management poses various challenges due to its composition of valuable elements like gold, copper, silver, and rare earth metals, as well as harmful substances such as cadmium, lead, and halogenated chemicals, which raise significant health and environmental issues [154,155]. These harmful substances can seep into the environment through traditional recycling processes like incineration or the leachate from landfills, highlighting the environmental implications of e-waste recycling, and many countries have started working on treating e-waste informally [156].

During incineration processes used for extracting copper from insulated cables or gold from printed circuit boards, there is a release of dangerous substances like furans, dioxins, and a range of other contaminants, illustrating the environmental dangers of e-waste treatment. Although there have been attempts to restrict informal recycling through laws and trade bans, the actual enforcement remains a significant challenge, highlighting the complexities in regulating e-waste activities [157].

Design for Disassembly (DfD) could be a valuable strategy to boost the recyclability of electronic devices, underscoring its role in promoting sustainable e-waste practices. For smart textiles, DfD is crucial for overcoming the composite material bottleneck by focusing on creating easily separable materials—specifically, designing the electronic modules for reversible detachment from the textile substrate to streamline the recovery of both the valuable and hazardous electronic fractions before the textile material is processed. This approach focuses on creating easily separable materials to streamline recycling, reusing, and maintaining electronic devices, thereby diminishing the environmental footprint. The ReUse project innovatively designed a printed circuit board that enables component disassembly by immersing it in hot water, showcasing a practical approach to promoting recyclability and sustainable design [158]. The success of these DfD strategies must be quantified through specific metrics, such as the calculated recyclability rate (the percentage of material mass recoverable in a secondary form) and the separation efficiency of the electronic fraction from the textile substrate. Product Service Systems (PSSs) offer an alternative approach by creating closed-loop systems where users exchange product components for replacements, illustrating their role in fostering sustainability and waste reduction [159]. The beverage industry has successfully implemented deposit refund mechanisms to reduce plastic waste, showcasing an effective strategy for managing waste and promoting recycling initiatives. Manufacturers of wearable gadgets could consider adopting similar strategies, like implementing trade-in programs, to incentivize and promote responsible disposal practices within the electronics sector [160].

**Figure 5:**
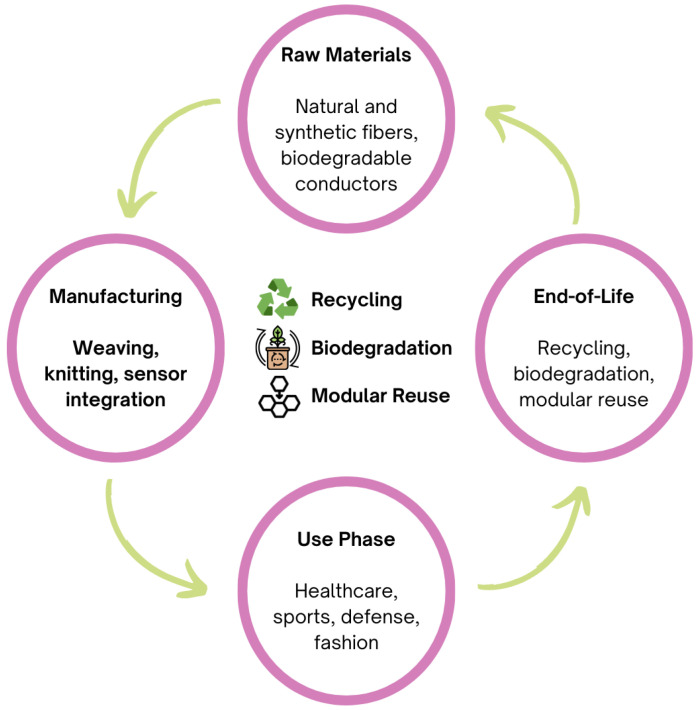
Lifecycle sustainability of smart textiles, illustrating a circular flow from raw materials and manufacturing through use and end-of-life. Central strategies—recycling, biodegradation, and modular reuse—are essential to achieving circularity and minimizing e-waste.

A holistic understanding of sustainability in smart textiles requires viewing the product not only through the lens of material choice or individual processes but across its entire life cycle. From the sourcing of raw materials to the integration of sensors during manufacturing, and from the user phase to end-of-life treatment, each stage presents opportunities to minimize environmental burden and implement circular strategies. Closing the loop through recycling, biodegradation, and modular reuse ensures that smart textiles contribute to reduced e-waste and resource efficiency rather than exacerbating existing challenges. Figure 5 illustrates this circular flow, emphasizing the interdependence of materials, processes, and recovery pathways that underpin sustainable design in next-generation smart textiles.

## 6. Future Directions

**Figure 6:**
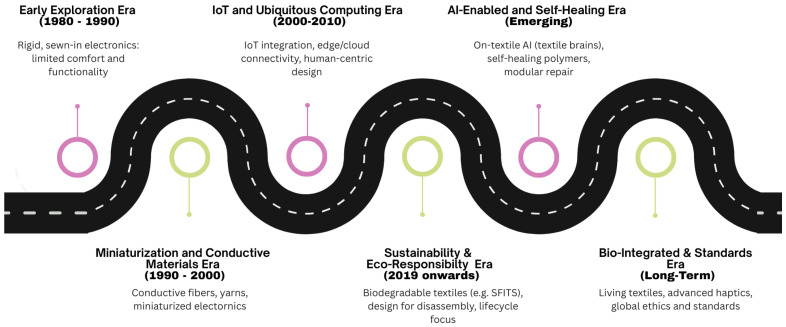
Roadmap of smart textiles evolution and future directions, highlighting the transition from early exploration with rigid devices toward miniaturization, IoT integration, sustainable materials, AI-enabled “textile brains,” and long-term visions of bio-integrated systems and global standards.

The evolution of smart textiles underscores a shift toward deeper integration of intelligence, sustainability, and human-centric design. Figure 6 illustrates this advancement, offering a concise overview of the field’s major milestones and emerging directions.

Building on this progression, several emerging and long-term research avenues are poised to shape the next generation of smart textile technologies. These directions encompass advances in artificial intelligence, materials engineering, modularity, bio-integration, interactive feedback, and regulatory frameworks, as outlined in the following subsections.

### 6.1. AI-Integrated Textiles and the Vision of “Textile Brains”

One of the most transformative directions is the integration of AI directly into textile systems [161]. On-device AI can enable real-time interpretation of physiological and motion data, providing personalized feedback and predictive capabilities without continuous reliance on cloud computing [6,162]. Recent innovations, such as Silk Fibroin Ionic Touch Screens (SFITS), already demonstrate how biodegradable interfaces can combine sensing, IoT connectivity, and AI-driven analytics within a single platform. Building on this trajectory, the concept of “textile brains” envisions distributed processing units embedded within fabrics that autonomously analyze signals, adapt to user needs, and communicate seamlessly with other devices [163]. Such systems could redefine human–technology interaction by placing intelligence closer to the body while safeguarding privacy and reducing latency.

In temrs of scientific challenges and research pathways, realizing the ’textile brain’ vision requires overcoming several technical bottlenecks. Key challenges include the development of ultra-low-power, textile-compatible processors capable of executing complex AI algorithms, such as Convolutional Neural Networks (CNNs), at the edge. Furthermore, research is needed to develop efficient distributed sensor fusion algorithms that can reliably integrate and interpret input from hundreds of heterogeneous sensors woven into the fabric. The issue of data and power management is critical; localized power sources must be sufficient for continuous inference, and the textile infrastructure must support high-speed, reliable internal data transfer between distributed nodes.

### 6.2. Self-Healing and Durable Fabrics

To achieve long-term usability, future smart textiles must withstand the rigors of stretching, abrasion, and repeated laundering. Self-healing polymers, adaptive coatings, and encapsulated conductors that autonomously repair micro-damages represent promising solutions [4]. These approaches not only extend functionality but also reduce waste by prolonging product lifespan. Establishing standardized washability and durability testing protocols will further accelerate industrial adoption.

### 6.3. Modular and Reconfigurable Architectures

Smart textiles should increasingly adopt modular and reconfigurable designs. Detachable sensing and processing units will enable customization, repair, and upgrades without discarding the entire garment, aligning with circular economy principles [1,3]. Design-for-Disassembly (DfD) strategies, such as standardized connectors and recyclable substrates, can facilitate scalable recycling systems and support sustainable business models like trade-in or component reuse.

### 6.4. Bio-Integrated and Living Textiles

An emerging research frontier involves bio-integrated or living textiles, incorporating natural fibers, microbial cellulose, or engineered organisms to achieve adaptive, self-sensing, or even self-regulating properties. Such systems could enable textiles that respond to biological signals or environmental conditions in real time [10]. However, these developments must be pursued with clear biosafety and ethical frameworks to ensure responsible innovation.

The primary hurdles in bio-integrated textiles center on stability and safety. Technical bottlenecks include ensuring the long-term viability and controlled function of biological components (microbes or enzymes) when subjected to wear, laundering, and body temperature fluctuations. Biocompatibility and regulatory biosafety are paramount, requiring new frameworks to ensure the integrated living materials do not pose risks to the user or the environment. Finally, achieving scalable and low-cost manufacturing methods for integrating biological matter into industrial textile processes remains a significant scientific challenge.

### 6.5. Advanced Haptics and Active Feedback

Smart textiles are moving beyond sensing into bidirectional interaction. The integration of distributed, low-power actuators will enable haptic feedback, tactile guidance, or proprioceptive cues. These capabilities hold potential for rehabilitation, sports training, and assistive navigation for visually impaired users [9]. Critical challenges include actuator miniaturization, energy efficiency, and human–factors research to ensure safe and intuitive feedback.

### 6.6. Standardization, Safety, and Ethics

The transition from laboratory prototypes to regulated products requires robust standards. Future efforts must focus on establishing metrics for accuracy, reliability, washability, and electromagnetic safety, ensuring dependable operation across use cases [3]. At the same time, ethical considerations—such as data privacy, informed consent, and equitable access—are paramount. Developing globally recognized frameworks for safety, sustainability, and responsible data management will be essential for building trust and accelerating adoption.

## 7. Conclusions

Smart textiles are entering a decisive phase where advances in conductive fibers, sensors, IoT frameworks, and AI integration are rapidly expanding their functionality and application domains. From healthcare and sports to defense, fashion, and emerging sectors, these fabrics promise to revolutionize human–technology interaction by providing continuous, adaptive, and unobtrusive sensing capabilities. A key insight of this review is that technical progress must evolve hand in hand with sustainability and human-centric design. Biodegradable systems such as SFITS illustrate how high functionality can be paired with environmental responsibility. Likewise, modular and reconfigurable architectures can reduce e-waste, while AI-enabled edge processing promises personalized insights delivered efficiently and privately. Together, these innovations highlight a path where smart textiles are both technologically advanced and ecologically sustainable.

Looking forward, several priorities must guide the field:

- Safety and Reliability: Ensuring robust operation under mechanical stress, repeated washing, and diverse environmental conditions, supported by standardized testing protocols.
- Sustainability and Circularity: Developing recyclable, biodegradable, and modular material systems to minimize environmental impact and promote circular business models.
- Ethical Considerations: Prioritizing data privacy, informed consent, and equitable access, ensuring that smart textiles respect human rights and societal values.
- Globally Inclusive Innovation: Promoting collaboration across regions to address diverse needs, avoid technological disparities, and ensure that benefits extend to communities worldwide.

In conclusion, the future of smart textiles lies not only in being “smart” but also in being inherently “sustainable” and “human-centric.” Achieving this vision will require interdisciplinary collaboration, transparent standards, and global cooperation. By aligning technological ingenuity with ecological and ethical responsibility, smart textiles can truly enhance human well-being and transform daily life in meaningful and inclusive ways.

## Figures and Tables

**Table 1:** Applications of Smart Textiles Across Industries.

Industry Sector	Example Smart Textile Application	Key Benefits	Sustainability Considerations	Challenges
Healthcare	Textile-based ECG electrodes, smart bandages for wound tracking and drug delivery.	Continuous monitoring, remote patient care, personalized medicine.	Biocompatibility, sterile disposal, energy efficiency.	Durability against washing/sterilization, high regulatory hurdles, data security.
Sports & Fitness	Garments capturing muscle activity and gait patterns.	Real-time feedback, personalized training, injury risk reduction.	End-of-life recycling (electronics/fibers), sustainable sourcing.	Sensor accuracy during activity, washability, component durability.
Defense & Security	Uniforms with physiological sensors, adaptive camouflage.	Enhanced soldier safety, operational efficiency, reduced equipment burden.	Ethical use, secure disposal, non-toxic materials.	Extreme environmental durability, reliability in isolation, power management.
Fashion & Wearables	Interactive fabrics (color/light), integrated wellness tracking clothing.	Functionality with aesthetics, enhanced user experience, comfort.	Design for disassembly (DfD), repairability, utilizing recycled/natural fibers.	Balancing trends with integration, achieving aesthetics with electronics, high production cost.
**Other** **Emerging** **Sectors**	Smart car seat fabrics, responsive furnishings, soft tactile skins for robotics.	Enhanced safety (e.g., driver fatigue), improved comfort, natural human-robot interaction.	Longevity and maintenance, efficient energy usage, reducing material waste.	Integration into existing systems, long-term reliability against factors (e.g., UV), scaling production.

**Table 2:** Comparative Evaluation of Communication Protocols for Smart Textile IoT.

Protocol	Range	Data Rate	Power Consumption	Security Features	Typical Applications
BLE	Short (up to 50 m)	Moderate (up to 2 Mbps)	Very Low	AES 128-bit encryption, connection key management.	Personal fitness tracking, real-time vital sign monitoring to a phone/hub.
Zigbee	Moderate (10–100 m)	Low (up to 250 kbps)	Low	AES 128-bit encryption, focus on network reliability.	Team sports monitoring, industrial worker health systems, home automation.
LoRaWAN	Long (km scale)	Very Low (0.3–50 kbps)	Ultra-Low	AES 128-bit for network and application layers.	Remote patient care, agricultural monitoring, asset tracking (low bandwidth).
Wi-Fi	Moderate (up to 100 m)	High (Mbps to Gbps)	High	WPA2/WPA3 encryption, direct IP connectivity.	Applications requiring high data throughput (e.g., video streaming) or direct internet access.
NFC	Very Short (cm scale)	Low (up to 424 kbps)	Very Low (Passive)	Secure element for authentication, proximity-based security.	Secure access control, textile authentication, quick data transfer on contact.

**Table 3:** Comparative Overview of Textile-Based Sensor Types.

Sensor Type	Material/Method	Key Performance Metric	Advantages	Limitations
Fiber-based	Conductive Polymers, Metal Nanowires, Coating/Spinning methods.	Flexibility, Gauge Factor (Strain), Sensitivity (Pressure).	Seamless integration, unrestricted movement, real-time monitoring.	Durability, washability, complex fabrication of functional fibers.
Yarns-Based	Composite yarns (e.g., BC/PCM/SS), Twisting, Ring Spinning.	Stability, Photoelectric Properties, Rectified Behavior.	Cost-effective manufacturing, maintains constituent functions, simplifies integration.	Requires specific yarn manufacturing (melt/solution spinning), limited sensor density.
Woven-based	Functional fabric, conductive yarns (e.g., Ag-plated Nylon), varied weaving patterns.	Mechanical Stability, Sensitivity Range, Interwoven unit combinations.	High mechanical stability, diverse pattern creation, adjustable sensor characteristics.	Sensitivity highly dependent on parameters (density, float length), insulation complexity.
Knitted-based	Conductive yarns (e.g., Ag-plated), 2D/3D Knitting, Intarsia technique.	Stretchability, Low Hysteresis, Linearity (Pressure).	High flexibility and stretchability, seamless large-scale integration, customizable 3D structures.	Loose structure can cause friction/hysteresis, requires optimization of knitting parameters.
Embroidery-based	Piezoresistive yarns, conductive thread on textile substrate, automated stitching.	Design Flexibility, Precision, Reproducibility.	High design flexibility and aesthetic appeal, cost-effective automation, customized placement.	Relies on substrate stability, potential wear at stitch points, limited to 2D patterns.
Substrate Modification	Coating (MXene, CNTs), LIG (Laser-Induced Graphitization), Deposition methods (CVD, Electrophoretic).	Coating Adhesion, Conductivity (Ω/sq), Sensing Stability.	Cost-effective for large areas, high conductivity potential, binder-free LIG methods.	Poor washability/durability, non-uniform coating on porous fabrics, energy-intensive processes (thermal carbonization).

## Data Availability

No new data were created or analyzed in this study.
